# The impact of Digitalized Communication on the effectiveness of Local Administrative Authorities – Findings from Central European Countries in the COVID-19 Crisis

**DOI:** 10.1007/s11573-022-01106-8

**Published:** 2022-07-14

**Authors:** Bernhard Hirsch, Fabienne-Sophie Schäfer, Aleksander Aristovnik, Polonca Kovač, Dejan Ravšelj

**Affiliations:** 1 Universität der Bundeswehr München, 85577 Neubiberg, Germany; 2 Faculty of Public Administration, University of Ljubljana, 1000 Ljubljana, Slovenia

**Keywords:** Local administrative authorities, COVID-19, Digitalization, Good governance, Comparative analysis, Central european countries, H12, K10

## Abstract

This paper analyses the impact of the use of digital communication tools in administrative procedures on the effectiveness of local administrative authorities during the COVID-19 pandemic. It considers COVID-19-driven changes in the legal competence of the institutional unit and administrative authorities’ orientation to good governance as mediators of this relationship. By applying partial least squares structural equation modelling (PLS-SEM) to survey data (N = 610) from five central European countries, we show that the accelerated use of digitalized communication tools driven by the COVID-19 situation has a positive effect on the effectiveness of local administrative authorities. Our data also indicate that the new laws, instructions and good governance driven by the COVID-19 situation – based on mostly convergent administrative traditions and European trends – partially mediate the relationship between the use of digital communication tools and administrative effectiveness. These findings do not significantly differ between participating countries and bureaucratic traditions. Consequently, the COVID-19 crisis proved to be a joint facilitator of responsive public governance that remained compliant with the rule of law, regardless of whether the national administrative systems were traditionally more legalistically or managerially oriented.

## Introduction

The COVID-19 pandemic has brought great and unforeseen challenges not only to societies at large but also to public administrations (Ansell et al. 2021; Schuster et al. 2020; Schomaker and Bauer 2020). During the pandemic, public administrations have been forced to provide public services differently, that is, “to alter the way they work and make decisions” (Phillips et al. 2021, p. 1). Consequently, the COVID-19 pandemic has challenged not only public leadership styles (Ansell et al. 2021; Dirani et al. 2020), but also public servants’ working routines and administrative cultures (Parker 2020; Gabryelczyk 2020).

More concretely, the pandemic has forced many public servants to work remotely (Gabryelczyk 2020) or at least “to move their operations online, wholly or in part” (Agostino et al. 2021, p. 69). This has induced the establishment of new procedures for providing services to external parties as well as different coordinating tasks inside administrations. In this context, the challenges of knowing how to handle and to distribute relevant information between public administrations and their ‘customers’, including citizens, firms and other parties, have become highly relevant (Phillips et al. 2021).

Already before the COVID-19 pandemic, the digitalization of public services was on the agenda of public administrations. Nevertheless, researchers have observed that COVID-19 has sped up the process of digitalization in public administrations, as many services for citizens, forms and other stakeholders had to be made available online during the lockdown (Hodžić et al. 2021; Gabryelczyk 2020). As a result, Agostino et al. (2021) speak about a “COVID-19-induced digital acceleration” (p. 69; similarly, Gabryelczyk 2020). The authors see this development as “an opportunity for scholars and practitioners to observe how governments and organisations have acted and reacted over a short period, providing important lessons for the future” (p. 69).

We contribute to this research by analysing the impact of the use of digitalized communication tools on the effectiveness of service provision by various European local administrative authorities in the COVID-19 pandemic. The digitalization of public sector communication with citizens, firms and other parties has been identified as “one of the cornerstones of the digitalization of the public sector” (Andersen et al. 2011, p. 439). In the current public management literature, the question of *how* information is processed and communicated by public administrations is important for responding adequately to the COVID-19 crisis (e.g., Phillips et al. 2021). Because little knowledge exists regarding how communication to citizens has been rearranged by local administrations, which are primary providers of services to citizens in the COVID-19 pandemic, we investigate the following research question: *How does the use of digitalized communication tools affect the effectiveness of public service provision by local administrative authorities in the COVID 19 pandemic?*

We focus on the service provision of local administrative authorities because they strongly affect the satisfaction of individuals and organizations with the state. Local administrative authorities typically provide services directly to their citizens (Fleischer and Carstens 2021). They interact very closely with individuals, local firms and other stakeholders and provide services that have a high impact on citizens’ and firms’ well-being or financial situation. For example, German counties are responsible for state-driven health care activities, provide buildings and infrastructure for schooling, and are responsible for building permissions and alien affairs. In Slovenia, local administrative authorities issue building permits, register residence, issue foreigners’ visas, grant traffic- and agriculture-related licences, etc. In Poland (districts and communities) and the Czech Republic (municipalities), the respective entities take care of service more than authority-oriented matters, such as taking care of local land use or healthcare.

The data for this study were collected in an international survey examining the impact of the first wave of the COVID-19 pandemic on local administrative authorities. The target population were public managers responsible for managing local administrative authorities that provide public services at the local level. This survey was disseminated to participants from the Czech Republic, Germany, Poland, Slovenia and Romania. All of these countries are member states of the European Union and share strong and similar (Germanic) administrative traditions and regulatory frameworks. Thus, they are a comparable group of countries. In contrast, other groups of European countries have completely different administrative traditions, especially Francophone and Scandinavian countries (Aristovnik et al. 2021; Bouckaert et al. 2020; Kuhlmann and Wollmann 2019).

The empirical data confirm the impact of digitalized communication of the service performance of public authorities in all countries but indicate no country effect. We thus contribute to the emerging literature on the consequences of the COVID-19 pandemic for administrative procedures conducted by public institutions. While previous literature has focused on consequences for either leadership, work organization or cultural aspects (Phillips et al. 2021; Ansell et al. 2021; Parker 2020; Gabryelczyk 2020), our study provides insights on the success of using digitalized communication tools in local administrative authorities. It confirms previous observations of a COVID-19 driven acceleration of digitalized procedures in public administrations. As we find a mediating effect of good governance orientation and COVID-19-driven new legislation on this relationship, our study is one of the first to provide statistically confirmed insights into how the “governance mechanism may support information acquisition and transformation processes” (Aben et al. 2021, p. 1145) in the public sector.

The paper is structured as follows. In Sect. 2, we derive a framework to determine whether and how the use of digitalized communication tools influences the effectiveness of local administrative authorities in terms of good governance. In Sect. 3, we describe our survey design and operationalize the different factors. In Sect. 4, we present the statistical results. Finally, we discuss the implications of our empirical findings (Sect. 5), consider the limitations of our study and offer suggestions for future research (Sect. 6).

## Theoretical framework and hypothesis development

### Administrative traditions as a framework for local administrative authorities’ work

Administrative traditions are an important factor in public administrations’ conduct and development. They incorporate various complex variables and are based on the historical development of a country or region, its culture, and the role of the state in society. An administrative tradition is a more or less enduring pattern in the style and substance of the public administration in a particular country or group of countries; it is seen as a composition of both ideas and structures (Painter and Peters 2010). Traditions include values and attitudes towards administrators and their attitudes towards citizens, the understanding of the rule of law, the economic system and prosperity or crises, the difference in power or authoritarian vs. participative orientation towards other stakeholders, the relations between politics and professionalism, law vs. management, (de)centralization of authorities, accountability relations, transition processes, etc. (ibid.).

In Germany, the bureaucratic-legalistic logic has a long tradition and is deeply rooted in the public sector (Meyer and Hammerschmid 2006; Pina et al. 2009). This logic has various characteristics that influence people, structures, and practices within public sector organizations. For example, good governance is ensured by the ‘Rechtsstaatsprinzip’ (“principle of legality”), which permits administrative activity exclusively on the basis of laws and results in a strong focus on the legal and procedural correctness of each administrative act. On a structural level, a bureaucratic-legalistic logic is characterized by strong hierarchical structures ensured by the ‘Weisungsprinzip’ (“principle of directives”), which means setting directives as the main governance mode (Meyer and Hammerschmid 2006).

In the Czech Republic, Poland and Slovenia, a classical continental European administrative culture exists in which “the legality principle and legal perspective have dominated the performance (efficiency) principle” (Bohata 2020, p. 22; similar Kovač et al. 2016; Ropret et al. 2018). Nevertheless, there are some evident differences, especially concerning the Central Eastern countries with a communist history (Kovač and Bileišis 2017). Here, Poland and the Czech Republic are part of the Visegrad group under major Soviet influence, while Romania not only experienced Soviet governance but was also under the influence of the French administrative state legacy. Slovenia, being rather independent of the Soviet Union despite the decades of socialism in former Yugoslavia, has continued to express a mixture of ‘Rechtsstaat’ tradition based on its Austrian-Hungarian history and post-socialist transitional processes. However, despite all these peculiarities, after 30 years of independence, especially for Slovenia and the Czech Republic and informally for Poland and Romania, strong convergence of administrative principles and governance can be observed based on these countries’ full membership of the EU (Hammerschmid et al. 2016).

A more in-depth examination of various elements in individual countries reveals that in some countries, public administration reforms are more top-down oriented than in Germany and other countries (see more in the national chapters in Kovač and Bileišis 2017). This can be attributed to the level of change accompanying the break with communism, as in Poland, Romania and the Czech Republic, or the small size of the country, as characteristic for Slovenia. The same can be identified for the administrative structures and level of (de)centralization. Recently, more managerially oriented approaches and rationalization of public funds have been introduced in Romania based on the New Public Management (NPM) model as in countries of rather Weberian traditions, such as Slovenia, the Czech Republic and to some degree, Poland. Thus, a focus on administrative procedures as a framework to primarily protect public interest above delivering individual rights is still much more relevant in Slovenia, Germany, and the Czech Republic than in Poland and Romania. On the other hand, the procedural focus usually contributes to higher transparency and accountability, albeit lower responsiveness, participation and efficiency.

These national differences are expected to influence the institutional settings of local administrative authorities, such as counties, communities or local administrative units.

### Administrative work and digitalized communication

According to Parker (2020), administrative work “can be operationalized through office design, staff working routines and normalized formal and informal behavioral customs” (p. 1945). In this study, we focus on changes in staff work routines, especially driven by the need to digitalize, in the COVID-19 crisis situation. The acceleration of digitalization in office procedures has been initiated by the health care challenges of the COVID-19 pandemic era: “As so many communities have been required or advised by the government to retreat to their homes and where possible, to work from home, this has brought with it major changes in the world of the office” (Parker 2020, p. 1952). More concretely, work from home and even in the office typically has to be done electronically by public servants, and the need to avoid personal contact with citizens, firm representatives and colleagues implies providing more services to the ‘customers’ of local administrative authorities digitally.

To provide public services, public servants typically need specific information on the ‘customer’ receiving the service. Information processing theory argues that a “difference in the amount of information required to perform the task and the information already possessed by the organisation” (Galbraith, 1973, p. 5) exists. As a consequence, public administrations have to deploy information-processing activities that best address this information asymmetry (Aben et al. 2021). With reference to Daft and Weick (1984), Aben et al. (2021) explicitly mention that these information-processing activities include “processing and communicating information” (p. 1149).

In the Covid-19 crisis, digital communication technologies “enable the connection of governments and people and facilitate governments’ attempts to prepare and implement policy decisions based on up-to-date data and information, which is a condition for efficient management of public funds for the provision of public services to all users” (Hodžić et al. 2021, p. 165; similarly, Kuhlmann and Heuberger 2021; Gabryelczyk 2020). Parker (2020) observes that teleworking improved the efficiency and effectiveness of public service provision during the COVID-19 pandemic. Similarly, Nam (2019) claims that effectiveness and efficiency are, among others, important attributes of government performance.

Accordingly, a local administration should ensure that the public services offered are available for citizens and other stakeholders (community) and meet their quality expectations (Grossi and Reichard 2008; Anderson and Young 1999). As a consequence, we focus on quality aspects of governance performance and use government effectiveness, defined as “perceptions of the quality of public services” (Kaufmann et al. 2010, p. 4), as the main performance driver of local administrative authorities in Central European countries.

Previous studies found positive effects of the use of communication technology by governmental institutions on government effectiveness (Nam 2019; Janssen and Estevez 2013; Hackney et al. 2007; Moon and Norris 2005; Norris & Moon, 2005; Eyob 2004).

Huber (1990) already proposed that the use of computer-assisted communication technologies “leads to higher quality decisions” (p. 64). In line with Parker (2020), we assume that COVID-19-driven advances in digitalization enhance the quality of public services provided by local administrative authorities. Therefore, we formulate the following hypothesis:

*H1: The COVID-19-accelerated use of digitalized communication tools by local administrative authorities has a positive impact on the effectiveness of these institutions.*

### Institutional pressure on administrative work and good governance

Neo-institutional theory assumes that structural constraints shape actors’ behaviour and that actors respond to isomorphic pressures from their institutional environments and adopt structures, practices, and routines that have high social value (Nitzl et al. 2020; Hiebl 2018). Therefore, organizations provide answers to external changes in expectations and formal rules (DiMaggio and Powell 1983; Oliver 1991).

In many European countries, clear principles about good governance exist (e.g., Aristovnik et al. 2018; Berg-Schlosser 2006; Berg-Schlosser 2004). Based on neo-institutional theory, we argue that such principles of good governance are still valid in a crisis situation, such as the COVID-19 pandemic, and still influence the behaviour of public servants in local administrations. Kovač et al. (2016) distinguish eight principles under good governance: rule of law (lawfulness), participation, consensus orientation, equity and inclusiveness, transparency, responsiveness, accountability, efficiency and effectiveness. The authors argue that all eight principles “should be applied together to make a complete whole” (p. 135). Nevertheless, the authors also emphasize that there exists a predominance of rule of law as the basis of the other principles, including participation and the effectiveness of authorities.

Good administration is seen as an element of good governance. It can be realized when “it strives – within good governance – for an efficient implementation of public policies and public interest” (Kovač et al. 2016, p. 136). Because most elements of good administration correspond to principles of good governance (Kovač et al. 2016), we can assume that good administration, in our context the efficient service provision of local administrations, is better achieved when principles of good government are respected in these administrations.

Regardless of whether these long-lasting principles of good governance are in evidence, in the COVID-19 situation, local administrations have been confronted with many new laws, guidelines and instructions, mainly issued from administrative bodies at the federal and national levels. For example, in March 2020, the Czech government declared a state of emergency and introduced a lockdown (Bohata 2020). In many European states, state governments “made tough decisions that restricted citizens’ freedoms in order to stop the spread of the virus” (Bouckaert et al. 2020, p. 768). New legislations were established to protect citizens better and to ensure public health. For example, Germany passed the Federal Infection Protection + Law (IfSG), and states adjusted their legislation addressing regional hospitals and public health institutions. We interpret these new laws and instructions as modified constraints of local authorities (similarly to Kuhlmann and Heuberger 2021) and expect them to influence the behaviour of the local administration’s employees as well.

Based on these arguments, we formulate the following hypotheses:

*H2a: The degree of COVID-19-driven new laws and instructions issued mediates the impact of the COVID-19-accelerated use of digitalized communication tools on the effectiveness of local administrations.*

*H2b: An orientation to good governance positively mediates the impact of the COVID-19-accelerated use of digitalized communication tools on the local administration’s effectiveness.*

**Fig. 1:**
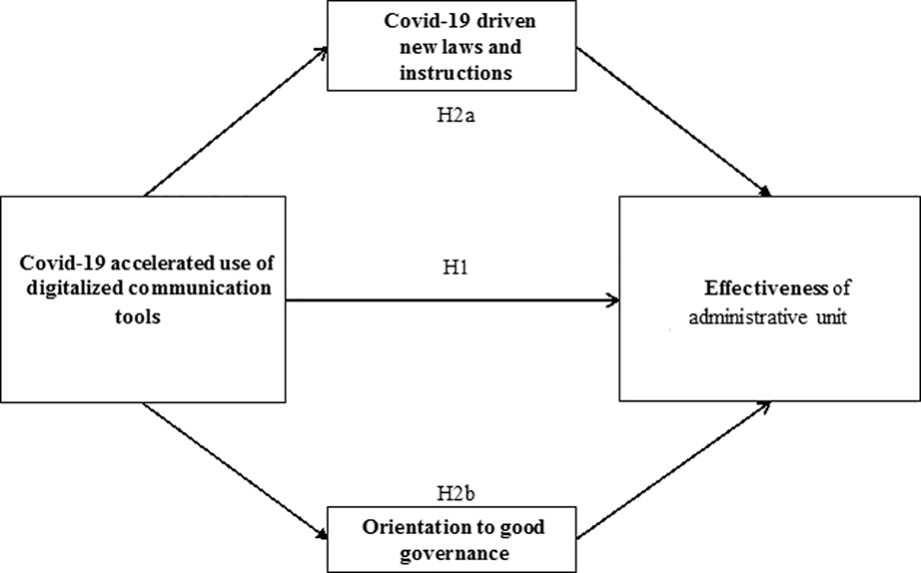
Research model

Figure 1 below shows our research model.

## Research design and method choice

The data for this study were collected in an international public administration survey examining the impact of the first wave of the COVID-19 pandemic on public administrations. The target population was public managers responsible for managing local administrative authorities that provide public services, such as general administrative territorial units on the local level. This survey was disseminated across participating countries. This analysis focuses on the Czech Republic, Germany, Poland, Slovenia and Romania. All of these countries are member states of the European Union and share strong (Germanic) administrative traditions. Thus, they are a comparable group of countries. In contrast, other groups of European countries have completely different administrative traditions, especially Francophone and Scandinavian countries (Aristovnik et al. 2021; Bouckaert et al. 2020; Kuhlmann and Wollmann 2019).

The online questionnaire was composed of 26 questions, including mainly closed-ended questions grouped into six parts (general, procedures and services, human resource management, economic and financial aspects, time after the pandemic, and demographic data). The questionnaire was translated into the national languages of the selected countries. The web-based survey was launched via the open-source web application 1KA (One Click Survey; www.1 ka.si) at the start of June 2020 and remained open until the end of August 2020, that is, in a period during which most of the nations covered experienced the arduous restrictions imposed by lockdowns.

The target population was public managers responsible for managing public administration authorities competent for conducting administrative procedures and providing public services as general administrative territorial units at the local level. The sampling technique used is non-probabilistic, convenience sampling facilitated by using information communication systems and channels. Participants were assured that the survey was strictly confidential and anonymous. The total sample consisted of 926 participants or public managers from five European countries (Czech Republic, Germany, Poland, Romania, and Slovenia), yielding a response rate of 51.8%. The response rate was different across countries and varied, with 27.2% in Germany and 28.0% in the Czech Republic, 58.2% in Romania and 66.2% in Poland, and even 100.0% in Slovenia. To prevent the overvaluation of counties with higher response rates, we used the country as a control variable. Considering the high hierarchical position of public managers, the response rates can be considered good (Hiebl and Richter 2018). To minimize potential bias, surveys with more than ten missing values or repeated answers were excluded, resulting in a final sample of 610 participants. In Table 1, we show more descriptive information about our sample.

**Table 1:** Sociodemographic and geographic characteristics of the survey respondents

Socio-demographic and geographic characteristics	Number (%)
**Years of employment in the current position in the administration**	
less than 1 year	40 (6.9)
1–5 years	185 (31.9)
6–10 years	127 (21.9)
11–15 years	94 (16.2)
16–20 years	59 (10.2)
more than 20 years	75 (12.9)
**Years of work experience**	
less than 10 years	35 (5.9)
11–20 years	142 (24.1)
21–30 years	237 (40.2)
31–40 years	175 (29.7)
**Size of local administrative authority (number of employees)**	
less than 20	45 (7.6)
21–40	89 (15.0)
41–60	38 (6.4)
61–80	5 (0.8)
81–100	122 (20.5)
more than 100	295 (49.7)
**Size of local administrative authority (number of inhabitants)**	
small administrative unit (up to 18.000 inhabitants)	121 (40.1)
medium administrative unit (18.000 to 50.000 inhabitants)	17 (5.6)
large administrative unit (50.000 to 100.000 inhabitants)	63 (20.9)
very large administrative unit (over 100.000 inhabitants)	101 (33.4)
**Coverage area of local administrative authority**	
predominantly urban area	210 (35.7)
predominantly rural area	379 (64.3)
**Country**	
Czech Republic	50 (8.2)
Germany	62 (10.2)
Poland	260 (42.6)
Romania	182 (29.8)
Slovenia	56 (9.2)

Note: The final sample consists of 610 respondents, some of whom did not provide information on sociodemographic characteristics

Using a survey to prove our hypotheses could be criticized, as the participants’ evaluations are subjective (Nitzl et al. 2020). Subjective measures are more responsive to common method variance (CMV) and could be affected by social desirability bias (Song and Meier 2018). Nevertheless, subjective measures are often the only way to receive internal information from organizations (Van der Stede et al. 2005; Speklé and Widener 2018). There are more reasons why using a survey is suitable for our study approach (Abernethy et al. 2013; Nicolaou et al. 2011; Schäfer et al. 2021). Walker and Andrews (2015) show that in a local government context, studies using archival data are not more robust in terms of overemphasized effects than those using survey data. Due to the complexity and length of the questionnaire employed in this study, the likelihood that the respondents would provide biased estimations based on cognitive maps is quite small (Chang et al. 2010).

We took additional measures to reduce CMV. We asked only public managers to participate in our survey study. Most of them had been working in the public sector for many years (approximately 60% of public managers had been in their position for more than 5 years, and approximately 70% of them had more than 20 years of work experience), and due to their function, they are expected to have good knowledge of the overall situation of their local administration. We assured them of anonymity to mitigate the possible effects of social desirability.

All constructs we measure are based on multiple items (Appendix A) and are measured on a 5-point Likert scale (1 = significantly smaller, 5 = significantly higher).^1^

The *COVID-19-accelerated use of digitalized communication tools* (“digitalized communication”) construct is based on the ideas of Moon and Norris (2005) and Andersen et al. (2011). Moon and Norris (2005) defined a construct called the “extent of municipal e-government” (p. 50), which contains variables for the adoption of municipal websites, the adoption of intranet and the development of a strategic e-government plan for municipalities. Similarly, Andersen et al. (2011) mention e-mail, web 2.0 tools, chats, video conferencing and webpage-oriented online services as dominant digitalization tools in public sector organizations. We adjusted these approaches to the COVID-19 crisis context, in which only short-term changes in the communication between civil servants of the local administration and its stakeholders (“parties”) could be realized. Therefore, we asked participants the following question: *During the pandemic period, compared to normal operations, please estimate the frequency of administrative procedures and services provided by the administrative unit in relation to parties through the communication channels listed below: (a) personal contacts (meetings, appointments, etc.) [DC_a], (b) phone [DC_b], (c) e-mail [DC_c], (d) administrative unit website [DC_d], (e) web portals (e-Government…) [DC_e], (f) video conferences (Zoom, Skype, etc.) [DC_f], and (g) social networks (Facebook, Twitter, etc.) [DC_g]. Communication via personal contacts [DC_a]* and *phone [DC-b]* are used as reverse items for measuring the use of digitalized communication contracts.

The construct *good governance orientation* (“good governance”) consists of five items. The items are based on the five most relevant principles of good governance, as described by Kovač et al. (2016). We asked the following question: *During the pandemic period, compared to normal operations, please estimate the orientation of legislation, guidelines and instructions (measures) to the following principles of good public governance: (a) rule of law [GG_a], (b) efficiency [GG_b], (c) responsiveness [GG_c], (d) participation [GG_4], and (e) accountability [GG_5]*.

To measure the degree of new laws and regulations that restrict the competence of a local organization (“new laws”*)*, we asked the participants to evaluate how strongly their working procedures were influenced by COVID-19-induced changes in legislation and other requirements. Such an approach is based on the theoretical understanding of the interdisciplinary operations of administration since purely legal or managerial measures do not suffice (Bouckaert et al. 2020; Mathis 2014). Any administrative conduct is legally determined since these affairs unilaterally affect citizens to primarily protect the public interest of the community. Moreover, the role of legislation is especially important for local entities that are obliged to equally implement national law. Inspired by the observation of Dzigbede et al. (2020) that “the list of executive orders issued by governors in response to the virus is breathtaking”, we asked the following question: *During the pandemic period, compared to normal operations, please estimate the following characteristics of legislation, guidelines and instructions (measures) defining the competences of the administrative unit: (a) inconsistency across areas/ministries [NL_a], (b) speed of adoption or enforcement [NL_b], and (c) abundant quantity – volume [NL_c].*

For a governmental context, effectiveness is defined as “a measure of the quality of [an]… output: how well did it achieve the desired outcome?” (Osborne and Gaebler 1992, p. 351). We operationalized (compared to Kovač et al. 2016, for instance) the *effectiveness* construct by asking about short term-oriented positive consequences accelerated by the COVID-19 situation for the administrations’ employees (digital competence, employee protection), the local administrations’ working processes (more efficient meetings, time savings) and “customers” (parties) (more digitalized and therefore faster-working processes, more completed tasks). In doing so, we address the specific quality aspects of governance output on a local level. We, therefore, asked the following question: *During the pandemic period, compared to normal operations, please estimate your perception of the employees’ opinions regarding the following positive consequences of the pandemic: (a) opportunity to digitize work processes - faster and more efficiently than in a normal situation [E_a], (b) opportunity to learn to use new digital communication tools (e.g., Zoom, MS Teams) [E_b], (c) opportunity to complete tasks that would have been difficult to complete in the previous situation [E_c], (d) having time to improve work processes [E_d], (e) more efficient meetings through digital communication channels [E_e], (f) awareness of the importance of workplace health promotion [E_f], and (g) awareness of the importance of protection for older employees and risk groups when organizing work [E_g].*

The hypotheses of our study assume cause and effect relationships. Structural equation models (SEMs) display such cause and effect relationships by showing the assumed relationships between latent constructs. SEMs allow the empirical testing of a causal model. To test complex models with direct and indirect effects and analyse collected data, Hair et al. (2017) proposed the use of partial least squares structural equation modelling (PLS-SEM). PLS-SEM is a variance-based approach of structural equation modelling. It allows the simultaneous estimation of construct measurements and structural path relationships. Accordingly, we use PLS-SEM to analyse our data and test the research model (see Fig. 1).

The respondents in our survey used the full range of possible responses from “significantly smaller” to “significantly higher” (1 to 5), which indicated the great difference perceived by the participants. The required sample size for detecting statistical power of at least 0.8 at an α-level of 0.05 is 77 (Nitzl 2016). Thus, with a sample size of 610, the relevant effects can be detected in our research model.

## Results

A SEM includes two parts: an outer measurement model and an inner path model. Latent variables, also known as constructs, are “the (unobservable) theoretical or conceptual elements in the structural model” (Hair et al. 2017). Constructs consist of a certain set of indicators, such as questions from a questionnaire. Arrows connect the indicators with their respective constructs, and the direction of the arrow shows their relationship (reflective vs. formative). These connections construct the measurement model (indicator – construct). The assumed relationships between the constructs make up the path model (Hair et al. 2017, 2019). The evaluation of the SEM follows a two-step approach. The first step includes the evaluation of the reliability and validity of a measurement model. This is the prerequisite for the second step. In the second step, the path model is analysed.

In the research model, only reflective measurements are used. Therefore, we used Cronbach’s α, composite reliability, AVE and HTMT to evaluate the reliability and validity of the measurement model (Hair et al. 2020). Items whose loadings were too low were dropped from the calculations; this was possible because the measurements are reflective. Table 2 shows that the adjusted constructs met all quality criteria, with one exception. The values for all loadings were above the critical value of 0.708. The AVEs for each construct were all above the critical value of 0.5, and all composite reliability values were above the critical value of 0.7. Just the Cronbach’s alpha for the construct “new laws” was below the value of 0.7. As Cronbach’s alpha generally underestimates internal consistency reliability in PLS-SEM, composite reliability provides a more appropriate measure in a PLS-SEM context (Nitzl 2016; Werts et al. 1974). Finally, HTMT indicated that all construct measurements are discriminant. Overall, all construct measurements in the research model are reliable and valid.

**Table 2:** Evaluation of the constructs

		Convergent Validity		Internal Consistency Reliability		Discriminant Validity
	**Indicators**	**Loadings**	**AVE**	**Cronbach’s α**	**Composite Reliability**	**HTMT**
**Critical values** ^**1**^		*> 0.708*	*> 0.5*	*> 0.7*	*> 0.7*	HTMT confidence interval does not include 1
Digitalized communication	*DC_c*	0.775	0.631	0.707	0.937	Yes
	*DC_d*	0.853				
	*DC_e*	0.752				
New laws	*NL_c*	0.902	0.724	0.627	0.839	Yes
	*NL_d*	0.797				
Good Governance	*GG_b*	0.875	0.809	0.766	0.894	Yes
	*GG_c*	0.923				
Effectiveness	*E_a*	0.767	0.625	0.802	0.870	Yes
	*E_b*	0.821				
	*E_f*	0.782				
	*E_g*	0.791				

^1^ Thresholds of the quality criteria need to be met. Each threshold is stated for each quality criterion

After the confirmation of the reliability and validity of the measurement model, the path model can be evaluated. There are two main criteria to evaluate the path model: the size of the path relationship and its significance. The interpretation of the path relationships is identical to the interpretation of regression coefficients. Table 2 summarizes the results of the path model and the relationship of the control variables to the focal construct “effectiveness”.

**Table 3:** Path coefficients and p values

	New laws	Good G = governance	Effectiveness
Digitalized communication	0.137**	0.234***	0.215***
Good governance	-	-	0.132**
New laws	-	-	0.171***
Years of employment^1^	-	-	0.021
Size inhabitants^1^	-	-	0.072
Size employees^1^	-	-	-0.037
Country^1^	-	-	0.068
Work experience^1^	-	-	0.000
Type^1^	-	-	-0.009
**R Square**	0.019	0.055	0.132
**R Square adjusted**	0.017	0.053	0.119

***, **, and * indicate significance at the 1%, 5%, and 10% levels (two-tailed)

^1^ Control variables

Table 3 illustrates that no control variable shows a significant effect (p-value < 0.05). Including the control variables in the research model means that other path relationships in the model are adjusted for the possible influence of a control variable.

The path coefficient of “digitalized communication” to “effectiveness” is highly significant (0.215; p < 0.01), which supports H1. In H1, we hypothesized that the COVID-19-accelerated use of digitalized communication tools by local administrative units has a positive effect on the effectiveness of the administrative units.

To assess the mediation effect, we follow Nitzl et al.’s (2016) and Sarstedt et al.’s (2020) approach by using PLS-SEM. According to Nitzl et al. (2016), mediation exists if the indirect effect is significant. If the direct effect is non-significant, full mediation is in place; otherwise, partial mediation exists. The results for the total, indirect, and direct effects, as well as the bias-corrected confidence intervals with a two-tailed significance level of 0.05, are presented in Table 4. If zero is not included in the confidence interval, this indicates that the effect is significant at the level of 0.05.

**Table 4:** Mediation analysis

Relations	Hypotheses^2^	Total Effect		Indirect Effects^3^		Direct Effect	
		**Coefficient**	**95% confidence interval** ^1^	**Coefficient**	**95% confidence interval** ^1^	**Coefficient**	**95% confidence interval** ^1^
Digitalized communication -> Effectiveness	H1	0.269	[0.173; 0.361]	-	-	0.215	[0.125; 0.300]
Digitalized communication -> New laws -> Effectiveness	H2a	-	-	0.023	[0.009; 0.044]	-	-
Digitalized communication -> Good governance -> Effectiveness	H2b	-	-	0.031	[0.014; 0.052]	-	-

Notes:^1^ If the interval does not include 0, the relation is significant.^2^ For mediation, the path coefficients of the respective paths are multiplied.^3^ Mediation exists if the indirect effect is significant. If the direct effect is significant, full mediation is in place. If the direct effect is nonsignificant, either complementary (positive paths) or competitive (negative paths) mediation exists

The statistical interpretation of mediation effects starts with the evaluation of the indirect effects, in our case, H2a and H2b. Because neither of the confidence intervals ([0.009; 0.044] and [0.014; 0.052]) include 0, we can confirm that the orientation to good governance (H2b) and the degree of new laws (H2a) positively mediate the relationship between the use of digitalized communication tools and the effectiveness of the administration. The confidence interval [0.125; 0.300] for the direct effect of “digitalized communication” on “effectiveness” also does not include 0. Hence, this direct relationship is partially mediated by “new laws” and “good governance”.

## Discussion

Local administrative authorities that conduct administrative procedures and provide public services should carry out their tasks with legal soundness and efficiency to comply with good governance principles. They are obliged by definition to search for an equilibrium between public interest protection and supporting the individual parties excising their rights in administrative relations (e.g., requiring various permits to run business). However, contemporary changes accompanying the digitalization of work highlight the need for more responsive and efficient ways of communication in public administration. Moreover, the COVID-19 pandemic, with the strong limitations of physical contact to minimize infections, requires public administration to continue to execute their competences and to be more flexible in their response to beneficiaries’ claims. In order to verify the main changes in the context of the administrative traditions in five selected central European countries regarding digitalization and good governance, the study was carried out based on the experiences of the first wave of COVID-19.

We confirm our initial hypothesis that the more intensive use of digitalized communication contributes to the efficiency of public service provision in all five central European countries. The impact of digitalized communication is significant and convergent and seems to last in local public services even after the first wave of the COVID-19 pandemic, as established by other studies (Bouckaert et al. 2020).

Our data indicate that COVID-19 not only brought new challenges to local administrative authorities but also gave them the opportunity to use digitalized communication tools more intensively. This has positive effects on the overall administrations’ effectiveness. In detail, the use of e-mail, the existence of a unit-specific website and work with web portals (with e-government) were significant indicators for the “digitalized communication” construct; all of them are channels that have been used by local administrations during the COVID-19 pandemic. In particular, websites and web portals are tools that were and still are typically used for communicating with the ‘customers’ of a local administration, i.e., citizens and other important stakeholders as parties to administrative procedures. This indicates that local administrators already offered digitalized services to their users, even when they were not legally obliged to do so. For example, the German Online Access Act (OZG) obliges not only the federal government and the states but also municipalities to deliver public services online by the end of 2022 (Fleischer and Carstens 2021). Therefore, our data from five Central European countries empirically strengthen the observation of Agostino et al. (2021) of a “COVID-19-induced digital acceleration”.

Our data also indicate that country-specific administrative traditions have no significant impact on the effectiveness of public institutions. Although we know from prior research that administrative traditions affect administrative structures and reform processes, we do not find any impact of country-specific or even regional traditions within our research model. This is rather surprising, as the corresponding literature argues that these traditions would lead to divergence in time and space (Mathis 2014; Kovač et al. 2016; Hammerschmid et al. 2016; Kovač and Bileišis 2017; Kuhlmann and Wollmann 2019). This legacy has been reasoned through historical institutionalism, political and administrative cultures or state traditions and patterns of governance. Previous studies have found that some countries favour more legally determined approaches with all policies enacted through legislation, and others prefer more flexible yet perhaps less certain public-legal relations when primarily pursuing principles such as efficiency within red tape removals and similar projects, i.e., continental *Rechtsstaat* and more authoritative versus participative approaches. We believe that there are three main explanations for why COVID-19 accelerated digitalization was not affected by these attributes. First, European legal harmonization and the convergence of administrative work has been relatively strongly developed over time both in Germany, a founding member since 1958, and in the other countries considered in this study, which are so-called “new” EU member states: the Czech Republic, Poland, and Slovenia all gained their full membership in 2004, while Romania joined in 2007. Second, the striving for globally acknowledged good governance in modern societies, encompassing not only legality in administrative relations but also responsiveness, participation, transparency and other contemporary principles, has been pursued by international and national authorities so systematically that the COVID-19 crisis not just suggested but required a deviation from former traditionally formalistic approaches. Third, our data indicate that during the pandemic, more and more users recognized the personal benefits of using digitalized communication tools. When asked whether they observed any increase in parties using digitalized communication, respondents in all countries observed a major change, especially in Slovenia (with a value of 4.6 in comparison to 3, indicating the same as before COVID-19), followed by Germany (4.3), Romania (4), the Czech Republic (3.9) and Poland (3.8). This indicates that digitalized communication tools – characterized as a form of “advanced information technologies” (Huber 1990) – may “overrule” country-specific communication traditions.

Following the above argumentation on the intertwining of several administrative principles, H2b is also confirmed, indicating that an orientation toward good governance positively mediates the impact of the COVID-19-accelerated use of digitalized communication tools on the local administration’s effectiveness. Our data show that this is a result of both institutional measures, especially changes in law and organizational improvement (“responsiveness”) and public servants’ behaviour regarding efficiency.

These findings indicate that none of the five countries participating in our survey study is governed by a single tradition or governance model, since individual models have shown various benefits but also weaknesses. Dysfunctions relate to politicization and an aggravated ‘ought’ perspective, leading to bureaucracy and formalism *per se* within Weberian concepts; a lack of constitutional state and erosion of democracy accompany NPM; and good governance enables relevant stakeholders to achieve collaborative governance only in a limited way and faces an outflow of democratic accountability due to the networking (Mathis 2014) governance model. These developments, along with top-down Europeanization (Hammerschmid et al. 2016; Kovač and Bileišis 2017) and crisis management, contribute to the convergence in public administration regardless of individual national traditions.

The procedures by which administrators issue general and individual legal acts are closely related to administration competences, especially in more Weberian and *Rechtsstaat* countries. Our data confirm H2a that the modified degree of new laws and regulations for local administrative authorities mediates the impact of the COVID-19-accelerated use of digitalized communication tools on the effectiveness of local administrative authorities. Naturally, during COVID-19, rule-making was fast-tracked and delegated from parliaments to the executive for timely action. We assumed that such rules might be inconsistent since there is usually not sufficient time to coordinate and contemplate legislation, which further hinders local administrative authorities from acting effectively. Nevertheless, our data show that the faster adaption of new laws, guidelines and instructions and the higher volume of new laws and guidelines passed due to the COVID-19 pandemic positively mediated the impact of the use of digitalized communication tools on the effectiveness of the local administrative authority. In contrast, the item measuring “whether new regulations and guidelines were inconsistent” was not significant. This means that the numerous new rules from higher authorities promoted the positive impact of using digital communication tools on the effectiveness of local administrative authorities. These rules were intended to enable local administrative authorities to act efficiently yet legally. In other words, the legislation issued by higher-level state institutions (e.g., sectoral ministries) was, despite the pressure to react quickly to the unforeseen pandemic, quite consistent and not ambiguous. As a consequence, it seems that governments, especially rule-making institutions, positively influenced the ability of local administrative authorities to work effectively during the pandemic. Moreover, it was shown that for any reform in public administration to be effective, it must also be legally grounded. In this sense, the law is not an obstacle to otherwise managerially led changes but a necessary guarantee to operationalize good governance, both regionally in central Europe and locally at the level of individual (types of) public administration.

The COVID-19 pandemic has revealed not only weaknesses and limitations of public administration but also hidden reform opportunities. The pandemic has created strong momentum for the digital transformation and reform of all aspects of public administration. Administrative procedures in particular, which are – due to the protection of public interest in administrative relations – conducted based on the rule of law and act as subsidiaries subjected to efficiency, can be further adjusted and developed. Namely, it seems that COVID-19-related digitalization showed how to bridge a collision between legality and responsiveness through an appropriate mix of formalism and flexibility. However, future studies need to establish how such a balance can also be reached in regular (non-pandemic) times so that individual parties to the procedure do not significantly misuse their entitlements. As the pandemic also raised questions related to the protection of citizens’ rights, privacy and personal data protection, further research could also consider these aspects.

## Conclusions

In this study, we showed that a COVID-19-accelerated, more intensive use of digitalized communication tools has a positive effect on the effectiveness of local administrations. Our data thereby indicate that public institutions are able to adapt administrative structures to quickly evolving challenges during a crisis situation (as proposed by Kuhlmann and Heuberger 2021). Second, our data indicate that fast-changing environmental factors, such as legislation adjusted due to COVID-19, have a positive impact on the relationship between the use of digitalized communication tools and the administration’s effectiveness. Law-makers seem to be able to set the ‘right’ rules to enable local administrations to offer services in a crisis situation.

Our results suffer from the classic limitations of survey-based studies (Nitzl et al. 2020). The conclusions are necessarily based on average evaluations. Hence, we provide an overview of the systematic trends, not idiosyncratic organizational factors. Our analyses are also based on the subjective evaluations of the respondents. This approach may lead to the misrepresentation of some key constructs in our survey. Our study is also focused on a specific point in time. A longitudinal study could analyse long-term changes in public service provision that are induced by the use of digitalized communication tools. Accordingly, future research could consider additional aspects that are beyond digitalized communication across selected segments of public administration, including local self-government and state administration. This would add a complement and comparative perspective to our study.

Nevertheless, according to our findings, we can sensitize politicians and public managers of local administrations to opportunities for digitalizing local administrations. In the future, when the COVID-19 pandemic has lost relevance, further steps of digitalizing public institutions should be implemented. This could include the adjustment of administrative processes and the reorganization of organizational structures, which both enable public administrations to use digitalized tools more intensively and effectively. Hence, the COVID-19 pandemic also brought a window of opportunity to accelerate necessary long-term public administration reforms.

## Footnotes

1 The abbreviations in square brackets, e.g., [DC_a] as the first indicator for digitalized communication, are used for naming the indicators of the constructs.
