# Correction to: The impact of Digitalized Communication on the effectiveness of Local Administrative Authorities – Findings from Central European Countries in the COVID-19 Crisis

**DOI:** 10.1007/s11573-022-01110-y

**Published:** 2022-08-16

**Authors:** Bernhard Hirsch, Fabienne-Sophie Schäfer, Aleksander Aristovnik, Polonca Kovač, Dejan Ravšelj

**Affiliations:** 1 Universität der Bundeswehr München, 85577 Neubiberg, Germany; 2 Faculty of Public Administration, University of Ljubljana, 1000 Ljubljana, Slovenia

## Correction to: Journal of Business Economics https://doi.org/10.1007/s11573-022-01106-8

Due to an unauthorized procedure in the publication process, there are errors in the original publication of this article. We apologize for these mistakes. The first error is in the second sentence in the fourth paragraph on page 7. The correct sentence is “With reference to Daft and Weick (1984), Aben et al. (2021) explicitly mention that these information-processing activities include “processing and communicating information (p. 1149).” The second error is the first sentence in the third paragraph on page 8. The correct sentence is “Huber (1990) already proposed the use of computer-assisted communication technologies “leads to higher quality decisions” (p. 64).”

In the Reference list two references are missing:

Galbraith, J.R. (1973), Designing Complex Organizations, Addison-Wesley Longman Publishing, Reading, MA

Huber, GP (1990): A Theory of the Effects of Advanced Information Technologies on Organizational Design, Intelligence, and Decision Making, The Academy of Management Review 15: 47–71

In the reference list the reference of DiMaggio et al. (1983) was displayed in an incorrect way. The correct reference is: DiMaggio, P., Powell, W.W. (1983): The iron cage revisited: Collective rationality and institutional isomorphism in organizational fields. American Sociological Review 48, 147–160

The original article has been updated.

